# Prevalence, molecular detection and risk factors investigation for the occurrence of *Toxoplasma gondii* in slaughter pigs in North India

**DOI:** 10.1186/s12917-019-2178-0

**Published:** 2019-12-03

**Authors:** Rashmi Thakur, Rajnish Sharma, R. S. Aulakh, J. P. S. Gill, B. B. Singh

**Affiliations:** School of Public Health and Zoonoses, Guru Angad Dev Veterinary and Animal Sciences University, Ludhiana, Punjab 141004 India

**Keywords:** India, Pigs, Prevalence, *Toxoplasma gondii*, Zoonosis

## Abstract

**Background:**

*Toxoplasma gondii,* an important food borne zoonotic parasite, infects almost all warm-blooded animals including pigs. People primarily become infected with *T. gondii* via consuming meat of infected animals. Status of *T. gondii* is largely unknown in pigs in India including northern regions. We, therefore, determined the prevalence of *T. gondii* infection in pigs from North India.

**Results:**

DNA of *T. gondii* was detected in 6.7% (54/810) of the tested slaughter pigs. Highest prevalence was observed in pigs from Punjab (8.2%) followed by Chandigarh (5.3%) and Uttarakhand (4.8%). Phylogenetic analysis revealed that the isolates from pigs had 96–100% nucleotide identity with Type I RH strain (AF179871), 96–99.7% with VEG type III strain (LN714499) and 67–72% with type II ME 49 strain (XM002370240). However, low level of polymorphism in the targeted B1 gene did not allow the determination of the clonal lineages of the isolates. Antibodies against *T. gondii* was reported in 48.3% (73/151) of the sera obtained from pigs slaughtered at Chandigarh abattoir, and scavenging by pigs was a significant risk factor.

**Conclusion:**

Prevalence of *T. gondii* DNA was low in pigs in North India, however, presence of the parasite warrants food safety concerns. Further studies are required to identify the clonal lineage of *T. gondii* circulating in pigs reared in North India. Pig farmers should be educated about the hygienic management practices.

## Background

Toxoplasmosis is caused by *T. gondii*, a zoonotic protozoan parasite which can infect almost all warm-blooded animals, including people. One third of human population worldwide has been exposed to *T. gondii* once in their life [16, 30, 31]. Infected people are usually symptomless or may have fever, malaise, myalgia, headache and lymphadenopathy [11]. Globally, 190,100 annual cases of congenital toxoplasmosis have been reported, leading to 1.20 million DALYs (Disability adjusted life years) and 1.5 cases of congenital toxoplasmosis per 1000 live births [40]. In India, 56,737 and 176,882 children are born with a possible risk of congenital toxoplasmosis every year [36]. Seroprevalence of 22% in pregnant women was reported [35]. First nationwide survey on *T. gondii* infection in humans in India conducted in 2005 indicated a seroprevalence of 24% (5611/23094) [9]. In North India, sero-prevalence was highest in Chandigarh (20%) followed by Uttar Pradesh (19%); prevalence was 13% in people of Punjab [9].

Animals play a pivotal role in the life cycle of *T. gondii* and its transmission to people. Felids are the only definitive hosts, which excrete oocysts of *T. gondii* in their faeces. Rodents and birds are the intermediate hosts in the natural life cycle of *T. gondii*; almost all warm-blooded animals (sheep, goat, cattle, pigs etc.) can also act as intermediate hosts. Intermediate hosts including people become infected via (1) consuming food and/ water contaminated with oocysts, (2) transplacental transmission (3) ingesting meat containing bradyzoites of *T. gondii* [16]. Ingestion of infected raw or inadequately cooked meat is considered a significant route of transmission in people. In a multicentric study in Europe, consumption of meat was considered as a potential risk of *T. gondii* infection [7]. It, therefore, becomes important to determine the status of *T. gondii* in the meat producing animals.

Several studies have been conducted in meat producing animals in India, and the prevalence varied from 1 to 85% [18, 22]. Only few studies detected the presence of *T. gondii* in pigs in India. Pigs are important for food security in India. As per the 19th livestock census, there are 10.29 million pigs in India [3]. The demand for pork has increased manifolds over the past few years. In India, most of the people involved in pork production belong to poor strata and thus, pork production is carried out under unhygienic conditions with low input costs exposing pigs to various infections [6]. Pigs reared are often allowed to roam freely and scavenge on unhygienic areas such as garbage disposal sites [6]. Such management practices can expose pigs to the zoonotic food borne parasites including *T. gondii.* To best of our knowledge, three studies have been conducted on *T. gondii* infection in pigs in India and two of them were based on serology i.e. detection of antibodies to *T. gondii* [4, 34]. Presence of anti *T. gondii* IgG in pigs indicates if they have been exposed to the parasite but does not tell about the presence of active infection, which becomes important especially for food safety concerns. Therefore, the primary aim of the current study was to detect DNA of *T. gondii* in the muscle tissues of pigs slaughtered for human consumption in North India. Previous research in pigs in India did not involve identification of the potential risk factors for the exposure to *T. gondii*. Such information is of utmost importance to develop and implement effective control strategies to combat food borne zoonotic parasites including *T. gondii*. To fill this knowledge gap, second aim of the current study was to investigate the seroprevalence of *T. gondii* and its association with the potential risk factors (for example age, sex and management practices) in the pigs slaughtered in an abattoir in Chandigarh.

## Results

### Prevalence of *T. gondii* in pigs from North India

DNA of *T. gondii* was detected in 54 of the slaughtered pigs indicating an overall apparent prevalence of 6.7% (95% CI = 5–9%) in North India. Prevalence was highest in province of Punjab (8.2, 95% CI = 6.0–11.2; 35/427) followed by Chandigarh (5.3, 95% CI = 2.7–10.1; 8/151) and Uttarakhand (4.7, 95% CI = 2.7–8.3; 11/232). District wise prevalence of *T. gondii* in pigs is provided in the Table 1.

**Table 1:** Prevalence of *T. gondii* in naturally infected pigs from North India

Location	Total number of samples	PCR positives	Apparent prevalence (%)	95%confidence interval (CI)
Punjab^a^	427	35	8.2	6–11
Ludhiana^b^	113	7	6.19	3–12
Jalandhar^b^	154	22	14.2	10–21
Bathinda^b^	90	4	4.44	2–11
Patiala^b^	70	2	2.85	1–10
Chandigarh^a^	151	8	5.29	3–10
Uttarakhand^a^	232	11	4.7	3–8
Udham Singh Nagar^c^	07	0	0	0–0.35
Nanital^c^	225	11	4.88	3–9
North India	810	54	6.67	5–9

^a^Province in North India

^b^Districts selected in Punjab

^c^Districts selected in Uttaranchal

### Sequencing and phylogenetic analysis

**Fig. 1:**
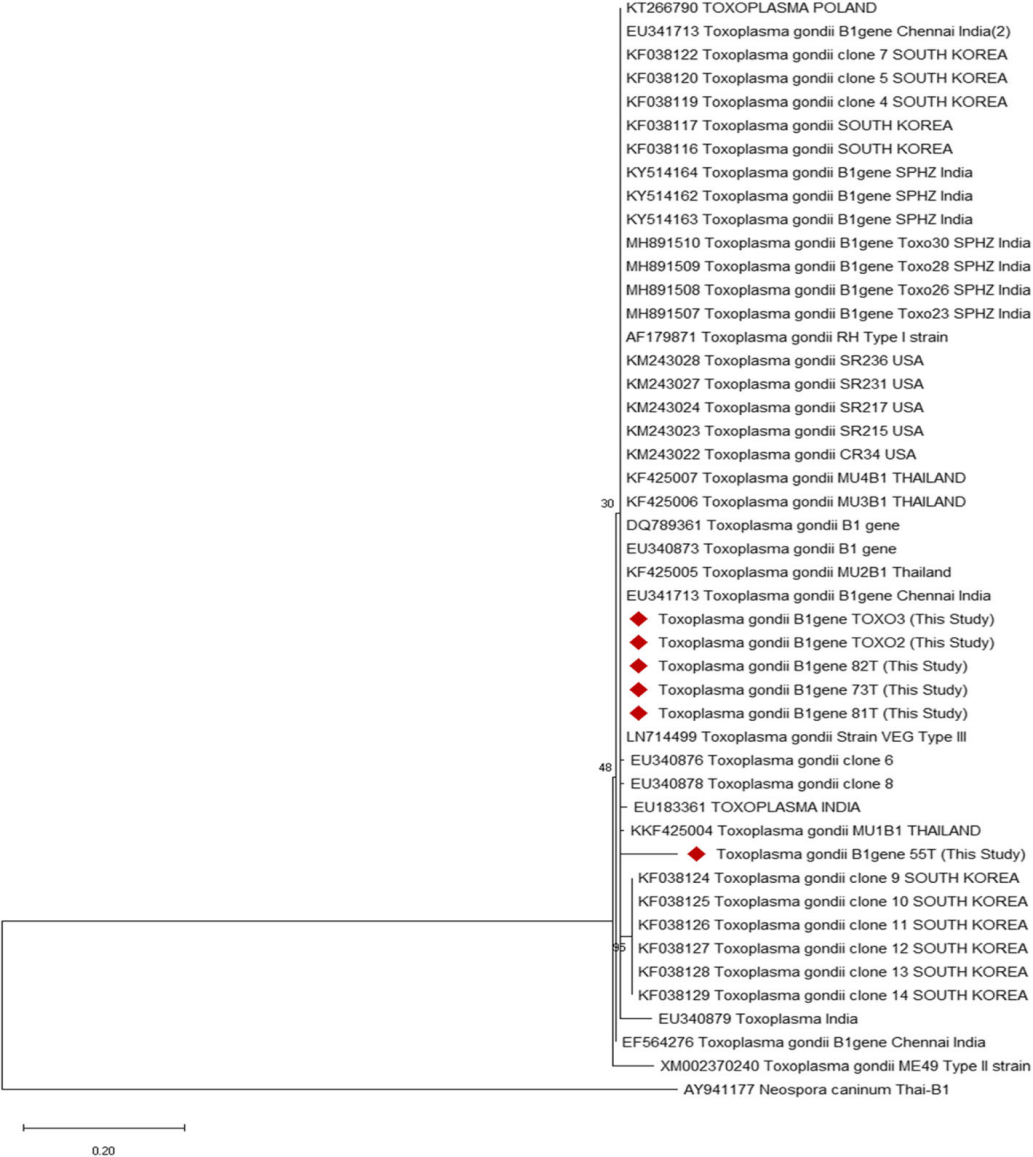
Phenogram construction of the B1 gene of *T. gondii* isolates from naturally infected pigs along with reference strains

The phylogenetic tree based on the alignment of B1 gene indicated that all positive samples except 55 T of the study were clustered with the VEG type III strain (LN714499) and unverified sequence EU341713 from Chennai, India; however sequence of positive sample 55 T of the current study clustered with unverified sequence KKF425004 MU1B1 Thailand (Fig. 1). The sequence alignment score of six sequences showed 96–100% nucleotide identity with Type I RH strain (AF179871) and VEG type III strain (LN714499) and 67–72% identity with Type II ME 49 strain (XM002370240). However, low level of polymorphism in the targeted B1 gene did not discriminate the clonal lineages of isolates identified in the current study.

### Seroprevalence and associated risk factors in pigs slaughtered at Chandigarh abattoir

Antibodies to *T. gondii* were detected in 73 (48.3, 95% CI = 40.5–56.3) of the 151 pigs tested; 3 animals showed doubtful results and were not included in further analysis. Antibodies to *T. gondii* were detected more frequently in pigs < 3 months (65%, 11/17) than pigs > 6 months (47%, 62/131). Almost equal proportion of males (50%, 11/22) and females (49%, 62/126) were anti- *T. gondii* antibody positive. Seroprevalence of *T. gondii* exposure did not differ significantly between age (Chi sq. = 1.818, df = 1, *p* = 0.178) and sex of pigs (Chi sq. = 0.05, df = 1, *p* = 0.945). Higher seroprevalence was reported in pigs that were allowed scavenging (58%, 73/125) than those that were not allowed; management practices were significantly associated with *T. gondii* exposure (Chi sq. = 26.5, df = 1, *p* = < 0.05).

### Comparison between PCR and ELISA

Out of 151 pigs tested for antibodies against (using ELISA) and DNA of (using PCR) *T. gondii*, 73 and 8 were positive, respectively. Of 8 PCR positives, all were ELISA positives, however, of 140 PCR negatives, 65 were ELISA positives. ELISA showed poor agreement (Kappa =0.11, 95% CI = 0.03–0.18) with PCR.

## Discussion

We document the first report of detection of DNA of *T. gondii* in pigs in India. Previously, DNA of *T. gondii* was detected in other meat producing animals (e.g. sheep, goat and poultry) from India [2, 27, 33]. Methods for detection of *T. gondii* or its DNA include bio-assays, tachyzoite culture, immunohistochemistry and PCR [29]; all but PCR are labour intensive and time consuming. PCR was used to detect *T. gondii* DNA in the tissues of pigs, but this method does not detect the viability of the parasite. Previously, viable *T. gondii* has been isolated from pork in Haryana, another north Indian state and elsewhere [14, 22, 32]. Further studies are required to isolate viable *T. gondii* in the pork samples destined for human consumption from North India. Overall, the presence of *T. gondii* DNA in the tissues of the pigs which were destined to human consumption presents a public health risk in North India. Hence, cooking pork adequately before consumption is highly recommended.

B1 gene, targeted for detection of *T. gondii* DNA in the current study, is a multi-copy sequence specific and highly conserved gene in all *T. gondii* strains [39], which makes it highly useful in molecular detection of parasite [37]. The phylogenetic analysis based on B1 gene showed clustering of positive samples with the VEG type III strain (LN714499) (Fig. 1). However, low level of polymorphism in B1 gene [28] did not discriminate the clonal lineages of different isolates obtained. Further studies are required to determine the genotypes of *T. gondii* circulating in the pigs of India.

A low prevalence of 7% recorded in the pigs in current study was comparable with the prevalence reported in sheep, goat (4–6%) and poultry (5%) in India [2, 27, 33]. This low prevalence could be attributed to false negative results due to use of small amount of tissue sample (200 ul) for extracting DNA in the current study. Low level of infection in pigs was reported in *T. gondii* infected pigs in a previous study, where one tissue cyst per 50 g of host tissue was detected [17]; however, the parasitic load was not quantified, presumably lower infection intensity in samples could be a possible explanation for low prevalence. Moreover, most common predilection sites for *T. gondii* in pigs include brain and heart followed by tongues; leg muscles have lower amount of the parasites ([19, 26]). Tongues or ham muscles were used for the detection of *T. gondii* in this study. Due to the afore mentioned reasons, prevalence estimated in the current study could be an under estimate of actual prevalence of *T. gondii* in the pork samples from north India, therefore further studies should use more sensitive techniques like magnetic-capture PCR ([26]) and common tissue predilection sites (brain and heart).

Surveys conducted in pigs globally recorded sero-prevalence varied from 0 to 92.7% ([5, 15];). High seroprevalence of *T. gondii* in pigs (48%) from Chandigarh was corroborated with other reports in pigs from India India and elsewhere [4, 32]. However, low seroprevalence was reported in pigs from Haryana, another north Indian state [34]; this variation in seroprevalences could be attributed to difference in the serological methodology, geography, management practices and sample size. Of 73 sero-positive pigs from Chandigarh, 6 were positive for the presence of *T. gondii* DNA, which indicated poor agreement between ELISA and PCR. Moreover, sero-prevalence of antibodies to *T. gondii* in pigs in India [15% in pigs from Haryana (northern state) [34], 32% in pigs in the North Indian [4], and 48% in pigs in current study] was higher than prevalence (of *T. gondii* DNA) in pigs in current study. Higher seroprevalence (of *T. gondii* antibodies) compared to prevalence (of *T. gondii* DNA) was expected as serology indicates exposure to pathogen not infection. On the other side, serology if detects IgG only can miss the acute infection and may lead to false negatives, therefore, a combination of molecular and serological tests can improve the diagnosis of *T. gondii*.

Scavenging by pigs was a potential risk factor for their exposure to *T. gondii*. Similar to our findings, higher seroprevalence was noticed in free ranging scavenging pigs in other parts of the world, e.g. Central Ethiopia, Netherlands, Zimbabwe [21, 25, 41]. Free ranging pigs have more exposure to *T. gondii* from the environment (contaminated food/water with oocysts, contaminated soil by rooting, infected meat scarps outside meat shops, consuming infected rodents). Previous studies in Brazil, USA and China found other potential risk factors for *T. gondii* exposure in pigs; for example, the presence of cats in or around farms, rodent control, carcass disposal and feeding leftovers [20, 24, 38].. Such information was not available for the pigs from Chandigarh; further epidemiological studies in pigs in India should also include these potential risk factors.

In India, 70% of the pig population is raised under traditional small holder, low-input demand driven production systems, except for limited number of semi-commercial pig farms in Kerala, Punjab and Goa [3]. In India, pig farming is largely done by the people with low socio-economic status [6]. Pigs are reared in poor hygienic conditions and have free access to garbage and stray animals (cats and dogs) or rodents, so they have higher chances of becoming infected with or exposed to *T. gondii*. Clinical manifestations are usually absent in pigs infected with *Toxoplasma gondii*, however have been reported especially in young pigs [10, 12, 15]. Reproductive losses (abortion and neonatal mortality) have been seen in sows infected with *T. gondii* [10, 12, 15]. Toxoplasmosis in pigs may lead to major economic losses to pig farmers. Therefore, pig farmers should be educated about good hygienic practices to raise healthy and disease-free pigs, which will help preventing economic losses as well as food borne zoonoses.

## Conclusion

The presence of *T. gondii* in pig meat from North India raises a public health concern, especially in free ranging scavenging pigs. Pig farmers should be educated about the hygienic management practices of pig farming. Cooking pork adequately before consumption is highly recommended. As infected meat producing animals including pigs are the direct source of infection to people, policies should be developed for routine screening of animals for *T. gondii*. Additional studies are necessary to isolate viable *T. gondii* from pork from India, and to determine the genotypes of *T. gondii* circulating in pigs.

## Methods

### Study area

The study area included Punjab (Latitude of 30°4′N and Longitude 75° 5′ E), Uttarakhand (Latitude of 30° 15′ N and Longitude 79° 15′ E) and Chandigarh (Latitude of 30° 44′14 N and Longitude 76° 47′ 14 E); all three regions are from North India. Uttarakhand, established as a new province in 2000, was carved from Uttar Pradesh (another North Indian state). There are 22 and 13 districts in Punjab and Uttarakhand, respectively. Agriculture contributes significantly to the economy of both these northern states. Chandigarh is a union territory and capital of Punjab. As per the 19th livestock census of India, there are 32,221, 19,907 and 132 pigs in Punjab, Uttarakhand and Chandigarh, respectively [3]. The pig slaughter is primarily conducted in small slaughter shops in Punjab and Uttarakhand. Abattoir in Chandigarh is the only pig abattoir in Punjab, where pigs from Punjab and neighbouring states are slaughtered.

### Target and study population

The target population primarily consisted of pigs slaughtered for human consumption in Punjab, Uttarakhand and Chandigarh. The study population included pigs slaughtered in four and two districts of Punjab and Uttarakhand, respectively as well as pigs slaughtered in Chandigarh abattoir. Six slaughter shops (one from each of the six districts [4 districts from Punjab: Jalandhar, Ludhiana, Bathinda, Patiala, and 2 districts from Uttarakhand: Udham Singh Nagar and Nanital]) were selected in Punjab and Uttarakhand; samples were collected from a pig abattoir from Chandigarh. The selection of shops in different districts or pigs in these shops was not random; however, we tried to ensure a loose representation by selecting slaughter shops in different areas and sampling on multiple occasions to ensure that pigs belong to different batches or pig owners.

### Sample size estimation

Estimated sample size was 669; it was calculated using Statulator [8] to estimate prevalence at 95% confidence, a design effect of 2%, and assumed prevalence of 32% based on a previous study [4].

### Collection of samples

Tongue or ham muscles from 810 slaughtered pigs were collected from the shops of Punjab and Uttarakhand, and from an abattoir from Chandigarh (Table 1). Age (< 3 months, > 6 months), sex (male, female) and management practices (allowed scavenging or not) for individual pig slaughtered at Chandigarh abattoir was also recorded. Samples were transported to the laboratory at − 20 °C.

### Pepsin- HCl digestion

The pepsin-HCl digestion was performed as per Dubey [13]. Briefly, 50 g muscle after removing fat and connective tissue was chopped into small pieces and grinded for 15 s at slow speed in a blender followed by addition of 125 ml of normal saline solution and blending at highest speed for 30 s. Homogenate obtained was transferred to a 1 L beaker and pre-warmed (37^0^ C; 250 ml) acid pepsin solution (pepsin 1:10000, HiMedia; 2.6 g, NaCl 5 g, HCl 7 ml and distilled water up to 500 ml) was added and incubated at 37^0^ C for 2 h. Homogenized solution was filtered through a sieve with a gauze and transferred to 50 ml plastic centrifuge tube. Centrifugation at 12,00 rpm for 10 min was performed and supernatant was discarded. Pellet was re-suspended in 5 ml of normal saline solution and stored at − 20 °C till further use.

### DNA extraction

DNA was extracted from 200 μl of re-suspended pellet using a commercially available kit (QIAamp DNA mini extraction kit, Qiagen, Netherlands) as per the manufacturer’s instructions and stored at -20 °C until further analysis.

### Nested- polymerase chain reaction (nested PCR)

To detect DNA of *Toxoplasma gondii*, primers amplifying B1 gene were used in a nested PCR assay as per previous protocol [23]. In each PCR assay, known positive (*T. gondii* DNA) and negative controls (nuclease free water) were used. DNA of *T. gondii* was provided by the Department of Parasitology, Post Graduate Institute of Medical Education and Research, Chandigarh.

### Phylogenetic analysis

PCR products of six positive samples were purified using Wizard® SV Gel and PCR Clean-Up System (Promega Biotech, India) as per manufacturer’s guidelines. The eluted DNA samples were sent for sequencing (Singapore). For analysing the sequencing results NCBI BLAST software was used [1] and the aligned dataset of B1 gene of *T. gondii* was analysed by Molecular Evolutionary Genetics Analysis (MEGA) software version X. The six B1 coding sequences (Toxo 2, Toxo 3, 55 T, 73 T, 81 T, 82 T) obtained in the current study were compared with the previously published B1 coding sequences [RH type I strain (AF179871), ME49 type II strain (XM002370240),VEG type III strain (LN714499)] and other 37 non-typing *T. gondii* B1sequences available in the GeneBank database. *Neospora caninum* B1 coding sequence of glycerol-3-phosphate dehydrogenase 1 (GPDH-1) similar to *T. gondii* was used as an out group for comparison. Phylogenetic tree was constructed with Maximum Likelihood (ML) method using Tamura-Nei model.

### Enzyme linked Immunosorbent assay (ELISA)

Using commercially available *Toxoplasma* ELISA kit (PrioCHECK® *Toxoplasma* Ab porcine), sera collected from pigs (*n* = 151) slaughtered in Chandigarh abattoir were tested for the presence of anti-*Toxoplasma* antibodies as per manufacturer’s guidelines.

### Statistical analysis

#### Prevalence and seroprevalence

Apparent prevalence and seroprevalences were calculated from the proportion of positive results of those tested by PCR and ELISA, respectively, and were presented with 95% confidence intervals.

#### Association between sero-status and risk factors

On the subset of pigs (pigs slaughtered at Chandigarh abattoir) that were tested for *T. gondii* antibodies, the associations between outcome variable (either seropositive/negative for *T. gondii* antibodies) and the predictors [age (< 3 months, > 6 months), sex (female and male) and management practices (allowed scavenging or not)] were determined using Chi square test. The doubtful results (*n* = 3) on ELISA were excluded in this analysis.

#### Comparison between PCR and ELISA

The kappa value (k) was calculated to determine the agreement between PCR and ELISA. Kappa values of ≤0.40, 0.40–0.60, 0.61–0.80 and ≥ 0.81 were considered to indicate slight to poor, moderate to good, substantial and excellent agreement, respectively [42]. All statistical analyses were performed using IBM SPSS (ver. 24; Armonk, New York, USA).

## Data Availability

The datasets used and/or analysed during the current study available from the corresponding author on reasonable request.

## Abbreviations

DALYs: Disability adjusted life years
ELISA: Enzyme linked Immunosorbent Assay
GPDH-1: Glycerol-3-phosphate dehydrogenase 1
MEGA: Molecular Evolutionary Genetics Analysis
ML: Maximum Likelihood
PCR: Polymerase Chain Reaction

## References

[1] Altschul SF, Gish W, Miller W, Myers EW, Lipman DJ (1990). Basic local alignment search tool. J Mol Biol.

[2] Anjali D, Vikrant S, Amit J, Amit S, Daya S (2017). B1 gene based semi nested PCR for detection of toxoplasmosis from poultry hearts. Ind J Anim Sci.

[3] Basic Animal Husbandry and Fisheries Statistics (BAHS) (2014). 19th livestock Census-2012, All India Report. Ministry of Agriculture Department of Animal Husbandry, Dairying and Fisheries, Government of India.

[4] Chhabra MB, Mahajan RC (1979). Occurrence of *Toxoplasma gondii* in slaughter pigs in India. Trop Geogr Med.

[5] Chandrawathani P, Nurulaini R, Zanin CM, Premaalatha B, Adnan M, Jamnah O, Khor SK, Khadijah S, Lai SZ, Shaik MAB, Seah TC, Zatil SA (2008). Seroprevalence of toxoplasma gondii antibodies in pigs, goats, cattle, dogs and cats in peninsular Malaysia. Trop Biomed.

[6] Chauhan A, Patel BHM, Maurya R, Kumar S, Shukla S, Kumar S (2016). Pig production system as a source of livelihood in Indian scenario: an overview. Int J Sci Environ Technol.

[7] Cook AJC, Gilbert RE, Zufferey J, Petersen E, Jenum PA, Foulon W, Semprini AE, Dunn DT (2000). Source of *Toxoplasma* infection in pregnant women: European multicenter case control study. Br Med J.

[8] Dhand NK, Khatkar MS (2014). Statulator: An online statistical calculator. Sample Size Calculator for Estimating a Single Proportion.

[9] Dhumne M, Sengupta C, Kadival G, Rathinaswamy A, Velumani A (2007). National seroprevalence of *Toxoplasma gondii* in India. J Parasitol.

[10] Dubey JP (1986). A review of toxoplasmosis in pigs. Vet Parasitiol.

[11] Dubey JP (1991). Toxoplasmosis--an overview. Southeast Asian J Trop Med Public Health.

[12] Dubey JP, Beattie CP (1988). Toxoplasmosis of animals and man.

[13] Dubey JP (1998). Refinement of pepsin digestion method for isolation of toxoplasma gondii from infected tissues. Vet Parasitol.

[14] Dubey JP, Hill DE, Jones JL, Hightower AW, Kirkland E, Roberts JM, Marcet PL, Lehmann T, Vianna MC, Miska K, Sreekumar C, Kwok OC, Shen SK, Gamble HR (2005). Prevalence of viable *Toxoplasma gondii* in beef, chicken, and pork from retail meat stores in the United States: risk assessment to consumers. J Parasitol.

[15] Dubey JP (2009). Toxoplasmosis in pigs- the last 20 years. Vet Parasitiol.

[16] Dubey JP (2010). Toxoplasmosis of animals and humans.

[17] Dubey JP, Lunney JK, Shen SK, Kwok OC, Ashford DA, Thulliez P (1996). Infectivity of low numbers of *Toxoplasma gondii* oocysts to pigs. J Parasitol.

[18] Dubey JP, Somvanshi R, Jithendran KP, Rao JR (1993). High seroprevalence of *Toxoplasma gondii* in goats from Kumaon region of India. J Vet Parasitol.

[19] Dubey JP, Murrell KD, Fayer R (1984). Persistence of encysted *Toxoplasma gondii* in tissues of pigs fed oocysts. Am J Vet Res.

[20] Feitosa TF, Vilela VL, de Melo LR, de Almeida Neto JL, Souto DV, de Morais DF, Athayde AC, Azevedo SS, Pena HF (2014). Toxoplasma gondii and Neospora caninum in slaughtered pigs from northeast, Brazil. Vet Parasitol.

[21] Gebremedhin EZ, Kebeta MM, Asaye M, Ashenafi H, Marco VD, Vitale M (2015). First report on seroepidemiology of *Toxoplasma gondii* infection in pigs in Central Ethiopia. BMC Vet Res.

[22] Gupta SL, Mahjan SK, Chhabra MB, Gautam OP (1982). Latent infection of *Toxoplasma* in pigs of Hissar, Haryana. Ind J Vet Med.

[23] Habibi GR, Imani AR, Gholami MR, Hablolvarid MH, Behroozikhah AM, Lotfi M, Kamalzade M, Najjar E, Esmaeil-Nia K, Bozorgi S (2012). Detection and identification of *Toxoplasma gondii* type one infection in sheep aborted fetuses in Qazvin Province of Iran. Iran J Parasitol.

[24] Hill DE, Haley C, Wagner B, Gamble HR, Dubey JP (2010). Seroprevalence of and risk factors for *Toxoplasma gondii* in the US swine herd using sera collected during the National Animal Health Monitoring Survey (swine 2006). Zoonoses Public Health.

[25] Hove T, Lind P, Mukaratirwa S (2005). Seroprevalence of *Toxoplasma gondii* infection in domestic pigs reared under different management systems in Zimbabwe. Onderstepoort J Vet Res.

[26] Jurankova J, Basso W, Neumayerova H, Balaz V, Janova E, Sidler X, Deplazes P, Koudela B (2014). Brain is the predilection site of *Toxoplasma gondii* in experimentally inoculated pigs as revealed by magnetic capture and real-time PCR. Food Microbiol.

[27] Kalambhe D, Gill JPS, Singh BB (2017). Molecular detection of *Toxoplasma gondii* in the slaughter sheep and goats from North India. Vet Parasitol.

[28] Khan A, Su C, German M, Storch GA, Clifford DB, Sibley LD (2005). Genotyping of *Toxoplasma gondii* strains from immunocompromised patients reveals high prevalence of type I strains. J Clin Microbiol.

[29] Liu Q, Wang ZD, Huang SY, Zhu XQ (2015). Diagnosis of toxoplasmosis and typing of *Toxoplasma gondii*. Parasit Vectors.

[30] Montoya JG, Liesenfeld O (2004). Toxoplasmosis. Lancet.

[31] Robert-Gangneux F, Darde ML (2012). Epidemiology of and diagnostic strategies for toxoplasmosis. Clin Microbiol Rev.

[32] Samico-Fernandes EFT, Samico-Fernandes MFT, de Albuquerque PPF, de Almeida JC, de Souza SA, da Rocha MA, de Souza Neto OL, Mota RA (2017). Toxoplasma gondii in backyard pigs: seroepidemiology and mouse bioassay. Acta Parasitol.

[33] Satbige AS, Bharathi MV, Ganesan PI, Sreekumar C, Rajendran C (2016). Detection of toxoplasma gondii in small ruminants in Chennai using PCR and modified direct agglutination test. J Parasit Dis.

[34] Sharma SP, Gautam OP (1974). A note on the prevalence of toxoplasma antibodies among camels and pigs in Hissar. Ind J Anim Sci.

[35] Singh S, Munawwar A, Rao S, Mehta S, Hazarika NK (2014). Serologic prevalence of *Toxoplasma gondii* in Indian women of child bearing age and effects of social and environmental factors. PLoS Negl Trop Dis.

[36] Singh S (2016). Congenital toxoplasmosis: Clinical features, outcomes, treatment, and prevention. Trop Parasitol.

[37] Switaj K, Master A, Skrzypczak M, Zaborowski P (2005). Recent trends in molecular diagnostics for Toxoplasma gondii infections. Clin Microbiol Infect.

[38] Tao Q, Wang Z, Feng H, Fang R, Nie H, Hu M, Zhou Y, Zhao J (2011). Seroprevalence and risk factors for Toxoplasma gondii infection on pig farms in central China. J Parasitol.

[39] Tavassoli M, Ghorbanzadehghan M, Esmaeilnejad B (2013). Foll Detection of *Toxoplasma gondii* in sheep and goats blood samples by PCR-RFLP in Urmia. Vet Res Forum Winter.

[40] Torgerson PR, Mastroiacovo P (2013). The global burden of congenital toxoplasmosis: a systematic review. Bull World Health Organ.

[41] Van der Giessen J, Fonville M, Bouwknegt M, Langelaar M, Vollema A (2007). Seroprevalence of *Trichinella spiralis* and *Toxoplasma gondii* in pigs from different housing systems in The Netherlands. Vet Parasitol.

[42] Viera AJ, Garrett JM (2005). Understanding interobserver agreement: the kappa statistic. Fam Med.

